# Successful Endoscopic Resection of an Early Carcinoma of the Duodenum

**DOI:** 10.1155/DTE.5.239

**Published:** 1999

**Authors:** Tomoko Tada, Jyunya Arai, Akihiko Ohta, Kouitirou Satoh, Katuya Hattori, Jun Ishiguro, Yoshinori Igarashi, Yoshihiro Sakai

**Affiliations:** Third Department of Internal Medicine Toho University 2-17-6 Ohashi Meguro-ku Tokyo 153-0044 Japan

## Abstract

We describe a patient in whom an early carcinoma of the duodenum was able to be resected endoscopically. A 77-year-old man presented with epigastric pain. Upper gastrointestinal endoscopy revealed a mass in the duodenum, and the patient was admitted. A whitish nodular aggregated lesion, measuring 20 mm in diameter, was found in the second portion of the duodenum. Examination of a biopsy specimen showed a Group III tubular adenoma. Endoscopic ultrasonography showed that the lesion was confined to the mucosa. The large size of the lesion suggested the possibility of malignancy. Endoscopic mucosal resection was therefore performed. Histopathologically, the diagnosis was carcinoma in adenoma. The depth of invasion was mucosal. We conclude that endoscopic muosal resection can be used to treat mucosal lesions arising in the duodenum.


*Diagnostic and Therapeutic Endoscopy*, Vol. 5, pp. 239–244
Reprints available directly from the publisher
Photocopying permitted by license only

© 1999 OPA (Overseas Publishers Association) N.V.
Published by license under
the Harwood Academic Publishers imprint,
part of The Gordon and Breach Publishing Group.
Printed in Malaysia.

### Successful Endoscopic Resection of an Early Carcinoma of the Duodenum

TOMOKO TADA*, JYUNYA ARAI, AKIHIKO OHTA, KOUITIROU SATOH, KATUYA HATTORI, JUN ISHIGURO, YOSHINORI IGARASHI and YOSHIHIRO SAKAI

*Third Department of Internal Medicine, Toho University, 2-17-6 Ohashi, Meguro-ku, Tokyo 153-0044, Japan*

*(Received 2 November 1998; In final form 23 March 1999)*

**We describe a patient in whom an early carcinoma of the duodenum was able to be resected endoscopically. A 77-year-old man presented with epigastric pain. Upper gastrointestinal endoscopy revealed a mass in the duodenum, and the patient was admitted. A whitish nodular aggregated lesion, measuring 20 mm in diameter, was found in the second portion of the duodenum. Examination of a biopsy specimen showed a Group III tubular adenoma. Endoscopic ultrasonography showed that the lesion was confined to the mucosa. The large size of the lesion suggested the possibility of malignancy. Endoscopic mucosal resection was therefore performed. Histopathologically, the diagnosis was carcinoma in adenoma. The depth of invasion was mucosal. We conclude that endoscopic mucosal resection can be used to treat mucosal lesions arising in the duodenum.**

*Keywords:* Early duodenal cancer, Endoscopic treatment, Indication of endoscopic mucosal resection, En block resection

### I INTRODUCTION

Primary duodenal carcinomas are rare. The estimated incidence ranges from 0.03% to 0.25% [1–3] of all patients undergoing autopsy and from 0.03% to 0.35% of all gastrointestinal cancers [4,5]. Recently, however, the number of reported cases has risen owing to the increased use of upper gastrointestinal endoscopy and radiography. In Japan some cases of duodenal carcinoma have been resected endoscopically [6–8]. Here we describe a case of carcinoma arising in the second portion of the duodenum that was able to be resected endoscopically.

### II CASE REPORT

The patient was a 77-year-old man. Angina pectoris had been diagnosed at 74 years of age, and he was receiving oral therapy. His family history was uncontributory. Since the end of March 1997, the

* Corresponding author.

patient experienced occasional epigastric pain. Upper gastrointestinal endoscopy revealed a mass in the duodenum. He was admitted to our department on April 25, 1997, for further evaluation.

On physical examination, a fist-size, pulsating mass (an abdominal aneurysm) was palpated in the upper umbilical region. There were no other abnormalities. Laboratory examinations showed no abnormalities except for mild anemia and renal impairment. Upper gastrointestinal endoscopy with a forward-viewing scope confirmed the presence of a mass in the second portion of the duodenum, but a frontal view could not be obtained; the position of the mass with respect to the papilla of Vater was unclear (Fig. 1). Observation was attempted a second time with a side-viewing scope, and we obtained a frontal view of a white nodular lesion measuring about 20 mm in diameter and located on the oral side of the papilla of Vater (Fig. 2(a)). The protrusion was enhanced by application of indigo carmine dye. The nodules were heterogeneous, and there was no distinct depression. There was no abnormality of the surrounding folds (Fig. 2(b)).

Hypotonic duodenography showed a clearly demarcated lesion in the posterior wall of the second portion of the duodenum. It showed a filling defect, consisting of aggregated flat nodules measuring

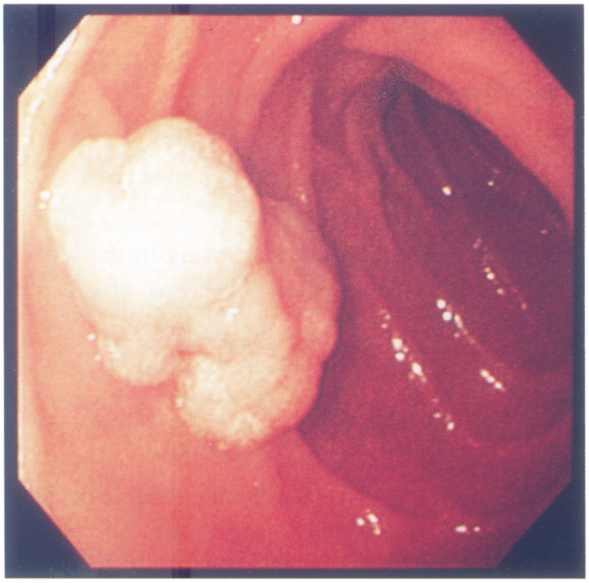

FIGURE 1 On examination with a forward-viewing endoscopy, a tumor was seen in the second portion of the duodenum. It could not be viewed in entirety.

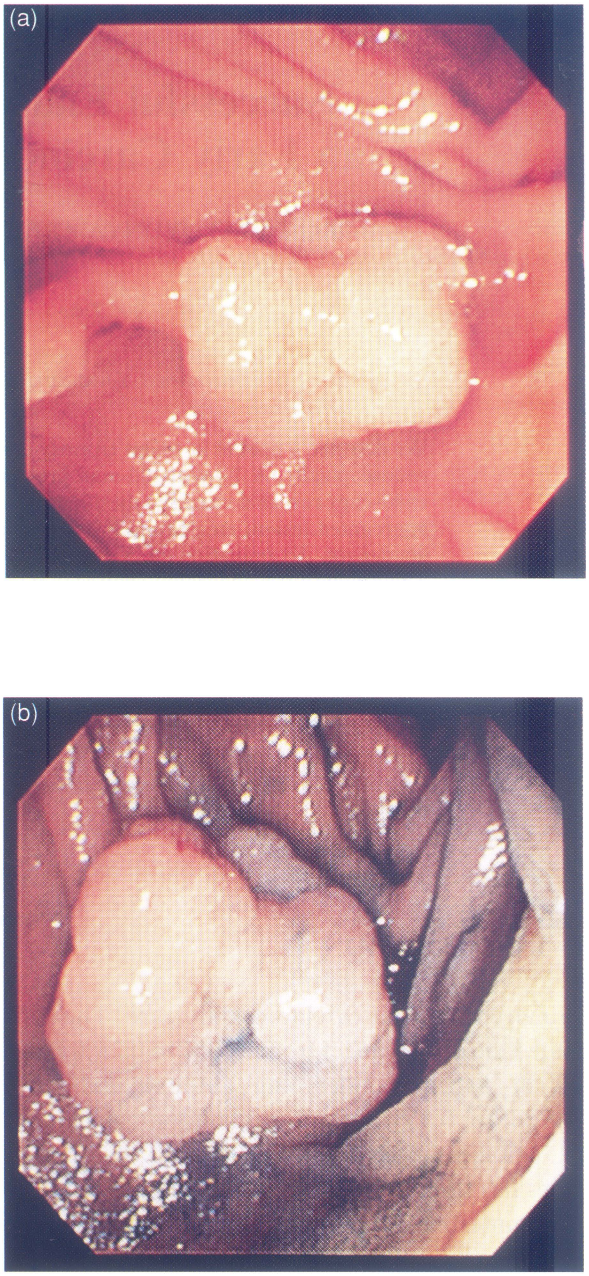

FIGURE 2 Examination with a side-viewing endoscope showed a white nodular lesion on the oral side of the papilla of Vater ((a) before dye spraying. (b) after dye spraying).

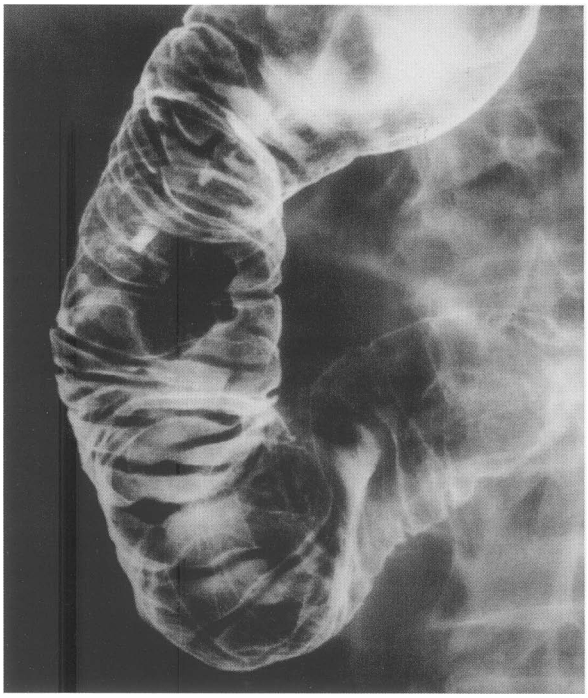

FIGURE 3 Hypotonic duodenogram showing a highly nodular lesion, measuring 22 × 18 mm in diameter, in the posterior wall of the second portion of the duodenum.

22 × 18 mm (Fig. 3). Endoscopic ultrasonography (EUS) with a small-diameter probe (UM-3R, Olympus, Tokyo, Japan) revealed thickening of the first and second layers and no change in the third layer, indicating a mucosal lesion (Fig. 4).

Diagnosis of histologic examination of a biopsy specimen was a tubular adenoma. The large size of the lesion suggested the possibility of carcinoma in adenoma. The lesion was therefore completely resected endoscopically on March 14. Before resection, an anticholinergic agent was administered to inhibit peristalsis. A direct-viewing double-channel scope (GIF-2T200, Olympus) was used to permit application of accessory instruments. To prevent puncture, physiological saline solution was injected locally to cause the lesion to protrude. The lesion was then resected with a crescent type snare. There was no postoperative bleeding or other complications.

On histopathological examination, the lesion measured 25 × 20 mm and was primarily a tubular adenoma. Only one portion consisted of well differentiated adenocarcinoma, showing nuclear and structural atypia (Fig. 5). The diagnosis was a mucosal carcinoma in adenoma with no lymphatic or vascular involvement. He died from rupture of abdominal aneurysm after 12 months. There was no evidence of recurrence till then.

### III DISCUSSION

Early cancer of the duodenum is generally treated by surgical resection. In Japan there is a recent trend toward endoscopic therapy not only by polypectomy but also by endoscopic resection [6–8]. To our knowledge, no report has described the use of endoscopic resection to treat duodenal cancer in Europe or North America.

Twenty-one cases of 22 lesions of early duodenal carcinoma, including ours, were treated by endoscopic resection and reported between 1993 and 1999 (Table I). The most common tumor site was the second portion of the duodenum. About half of the cases were diagnosed preoperatively as cancer, which is higher than reported previously but still low. Mean tumor diameter was 12 mm (range, 4–30 mm). Three cases (19, 20, 30 mm) resected piecemeal. In our patient, the lesion diameter was 25 mm, longer than that of any previously en block resected case described in the literature. Histopathologically, most duodenal cancers are well differentiated type adenomas [9]. Among the 23 previously reported lesions, the depth of invasion was mucosal in 19 and submucosal in 3. Surgery was additionally performed in 1 of the 3 patients with submucosal tumors, but remaining 2 patients refused surgery and were followed up with no further treatment [8,10]. Early duodenal carcinomas show no correlation between maximum tumor diameter and the depth of invasion. Duodenal perforation accidentally occurred in Cases 6 [11] and 13 [12]. One of these cases responded to conservative therapy and laparotomy was performed in the other. There was no report of recurrence.

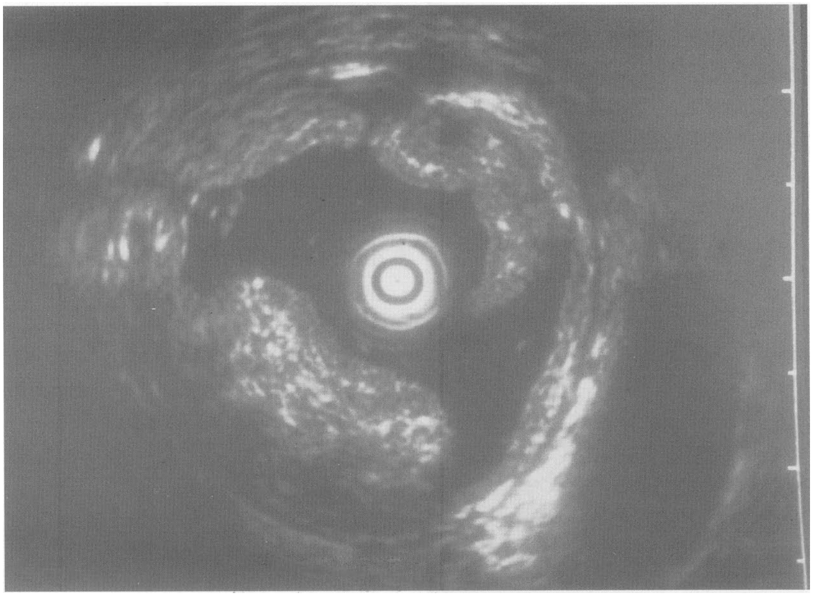

FIGURE 4 Endoscopic ultrasonogram showing thickening of the first and second layers. The third layer was intact.

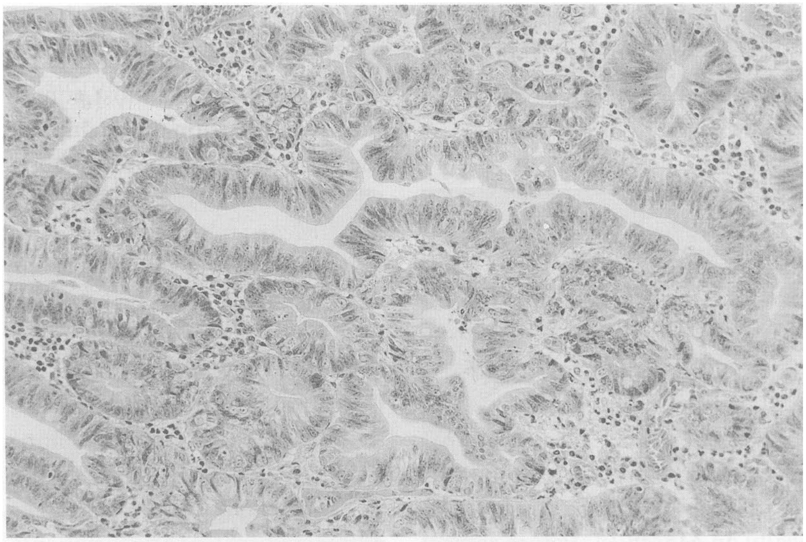

FIGURE 5 Histological photograph, showing well differentiated adenocarcinoma.

In primary duodenal carcinoma, it is difficult to differentiate benign from malignant tumors solely on the basis of endoscopic findings and, as noted above, biopsy specimens yield a low rate of positive diagnosis. Furthermore, since early duodenal cancer shows no correlation between tumor diameter and invasion depth, endoscopic resection to allow biopsy of the entire lesion should be performed when endoscopic ultrasonogram and other examinations indicate only mucosal invasion, irrespective of tumor diameter. However, there are several problems with endoscopic treatment of lesions in the

TABLE I Reported cases of early duodenal cancer terated by ER

| No. | Year | Age | Sex | Part | Biopsy | Type | EUS | Size (mm) | Histology | Depth | follow up | recurrence |
|---|---|---|---|---|---|---|---|---|---|---|---|---|
| 1 | 1993 | 66 | M | 2 | 5 | IIa | M | | Well | m | | |
| 2 | 1993 | 42 | M | 2 | 3 | IIc | | 6 x 3 | Well | m | 11M | — |
| 3 | 1993 | 61 | M | 1 | 5 | IIa | | 4 | Well | m | | |
| 4 | 1995 | 56 | M | 2 | 5 | IIa + IIc | M | 15 | Well | m | 6M | — |
| 5 | 1995 | 72 | M | 2 | 5 | Is | M | 8 x 6 | Mode. | sm | 10M | — |
| 6 | 1995 | 72 | F | 1 | 3 | Is | | 8 x 5 | Well | m | | |
| 7 | 1995 | 61 | M | 2 | | IIc | | | Adenocarcinoma in adenoma | m | | |
| 8 | 1995 | 79 | M | 2 | | IIc | | | Adenocarcinoma | m | | |
| 9 | 1995 | 63 | M | 1 | | Is | M | | Well | sm | | |
| 10 | 1996 | 52 | M | 2 | 3 | IIc + IIa | | 8 x 8 | Well | m | 6M | — |
| 11 | 1996 | 66 | M | 1 | 5 | Ip | | 12 | Adenocarcinoma | m | | |
| 12 | 1996 | 50 | M | 1 | | IIc + IIa | | 8 | Well | m | | |
| 13 | 1997 | 48 | M | 3 | 4 | IIc + IIa | | 7 x 7 | Well | m | 8M | — |
| 14 | 1997 | 67 | F | 2 | | Ip | | 19 | Well | m | | — |
| 15 | 1997 | 70 | M | 2 | | IIa + IIc | | 20 | Well | m | | — |
| | | | | 2 | | IIa + IIc | | 8 | Well | m | | — |
| 16 | 1997 | 85 | M | 2 | | I sp | | 20 | Well | m | | — |
| 17 | 1997 | 76 | M | 1 | | I sp | | 10 | Well | m | | — |
| 18 | 1997 | 44 | M | 2 | | I s | | 12 | Well | m | | — |
| 19 | 1997 | 61 | F | 1 | | I p | | 8 | Well | m | | — |
| 20 | 1998 | 65 | M | 1 | 5 | I sp | | 30 x 20 | Adenocarcinoma | sm | 2Y8M | — |
| 21 | Our case | 77 | M | 2 | 3 | IIa | M | 25 x 20 | Adenocarcinoina in adenoma | m | 12M | — |

Part – 1: bulb, 2: second portion of the duodenum, 3: third portion of the duodenum.
Biopsy – 3: adenoma, 4: adenocarcinoma suspect, 5: adenocarcinoma.
Histology – Well: Well differentiated adenocarcinoma, Mode: Moderate differentiated adenocarcinoma.

duodenum. First, the duodenal wall is thin, creating a high risk of perforation. Second, the lumen is narrow, and peristalsis occurs frequently. The scope is therefore less maneuverable than when placed in the stomach, and it is difficult to maintain an adequate field of view. Third, after resection the tumor tends to move anally, and large tumors are difficult to pass through the pyloric sphincter and retrieve. In our case, the tumor was located in the posterior wall of the second portion of the duodenum. A frontal view was difficult to obtain with a direct-viewing scope. After the injection of physiological saline solution, however, the tumor could be viewed frontally, facilitating complete resection. In the event that a direct view could not be obtained, we used a two-channel scope, which allows the use of a grasping forceps. To assess the anal side of the lesion and the spatial relationship with respect to the papilla of Vater, it is essential to also use a side-viewing scope to examine the lesion. It is also important to inhibit peristalsis by administration of an anticholinergic agent immediately before endoscopic resection.

Theoretically, endoscopic resection is indicated for the treatment of mucosal lesions with no lymph node metastasis. Indications for endoscopic treatment of early duodenal carcinoma have not been established. Nagatani reported that the rate of lymphonode metastasis is 0% for intramucosal cancers and 5% for submucosal cancer [13]. Hirasawa reviewed Japanese literature and reported that lymphonode metastasis did not occur when protruding or elevated tumors were less than 50 mm in diameter, so these lesion could be removed completely [14]. On the other hand, in depressed type, submucosal invasion has not been observed in lesions under 10 mm. So indication of depressed type early duodenal carcinoma has reported under 10 mm in size [12,14]. Other organs, the diameter of differentiated type mucosal carcinomas of the stomach ranges from 15 to 30 mm [15] and is generally considered to be within 20 mm. The size of lesions that can be resected en block is limited, and in the colon [16] lesions 20 mm or less lie within the safe zone, those 25–30 mm are in the borderline zone, and those more than 30 mm require piecemeal resection or laparoscopic surgery. The size of the lesion in our patient, i.e., 25 mm, is thus considered the upper limit for endoscopic resection of duodenal lesions. Some recommend piecemeal resection to permit larger lesions to be resected endoscopically, and careful planning of the strategy for piecemeal resection is useful in ensuring an adequate resection margin [17]. One problem is that, similar to other organs, tumor reconstruction is difficult, and traces of tumor may remain at the resection margin. In fact, the complete resection rate is low for piecemeal resection [15]. As mentioned previously, the anatomic characteristics and folds of the duodenum may preclude piecemeal resection. However, the number of patients in whom endoscopic resection is relatively indicated will probably increase, and if current problems are overcome, endoscopic resection, even when performed in a piecemeal fashion, will most likely be performed more frequently.

We conclude that by carefully determining the technique of choice, endoscopic resection can also be used to treat duodenal lesions.

### References

[1] Hoffman, W.J., Pack, G.T. *et al.* Cancer of the duodenum. *Arch. Surg.* 1937; **35**: 11–65.

[2] Sarma, D.P., Weilbacher, T.G. *et al.* Adenocarcinoma of the duodenum. *J. Surg. Oncol.* 1987; **34**: 262–263.

[3] Burgermamm, A., Baggentoss, A.H., Cai, J.C. *et al.* Primary malignant neoplasma of the duodenum excluding the pappila of Vater. *Gastroenterology* 1956; **30**: 421–431.

[4] Barcly, T.H.C. and Kent, H.P. Primary carcinoma of the duodenum. *Gastroenterology* 1956; **30**: 432–436.

[5] Lillemorek and Imbembo, A.L. Malignant neoplasms of the duodenum. *Surg. Gynecol. Obstet.* 1980; **150**: 822–826.

[6] Ooyama, T., Sakurai, H., Okada, M. *et al.* A case of early duodenal cancer completely resected by endoscopic mucosal resection. *Prog. Dig. Endosc.* 1996; **48**: 196–197.

[7] Sakane, M., Kashiwagi, R., Mitsutsuji, M. *et al.* A case of depressed type early duodenal carcinoma treated by endoscopic mucosal resection. *Dig. Endosc.* 1996; **8**: 231–235.

[8] Tutida, K., Itou, M., Takeuti, T. *et al.* A case of submucosal duodenal cancer excised by endoscopic mucosal resection. *Gastroenterol. Endosc.* 1995; **37**: 998–1003.

[9] Heniford, B.T., Iannitti, D.A., Evans, P. *et al.* Primally nonampullary/periampullary adenocarcinoma of the duodenum. *American Surgeon* 1998; **64**: 1165–1169.

[10] Ando, T., Ueda, M., Hongo, H. *et al.* A case of double cancer of the stomach and duodenum resected by endoscopy. *Gastroenterol. Endosc.* 1998; **40**: 779–785.

[11] Ohno, K., Hirasawa, Y., Yamamoto, K. *et al.* A case of early duodenal cancer treated by endoscopic mucosal resection. *ENDOSCOPIC FORUM for digestive disease* 1994; **10**: 262–265.

[12] Ogino, H., Fuzisawa, T., Sakashita, M. *et al.* A case of depressed type early duodenal cancer. *Gastroenterol. Endosc.* 1997; **29**: 1089–1093.

[13] Nagatani, K., Takekoshi, T., Baba, Y. *et al.* Indications for endoscopic treatment of early duodenal cancer: based on cases reported in the literature. *Endosc. Digest.* 1993; **7**: 969–976.

[14] Hirasawa, R., Ishii, H., Tatsuta, M. *et al.* Resection of duodenal adenocarcinomas, adenomas with submucosal saline injection. *Gastrointestinal Endosc.* 1997; **46**: 507–513.

[15] Hamada, T., Kondou, K., Itagaki, Y. *et al.* Endoscopic mucosal resection for early gastric cancer, limitation of whole block resection and problem of partial resection. *Stomach and Intestine* 1996; **31**: 1073–1082.

[16] Tamegai, Y., Ohba, H., Komatubara, N. *et al.* Indications and limitation of endoscopic treatment for early colorectal cancer. *Prog. Dig. Endosc.* 1997; **50**: 86–91.

[17] Tani, M., Inoue, H., Kndo, F. *et al.* Endoscopic mucosal resection for early gastric cancer – Usefulness of planning fractionated resection. *Prog. Dig. Endosc.* 1995; **47**: 64–67.

